# Complete Genome Sequence of the *Autographa californica* Multiple Nucleopolyhedrovirus Strain E2

**DOI:** 10.1128/genomeA.01202-14

**Published:** 2014-12-11

**Authors:** Ajay B. Maghodia, Donald L. Jarvis, Christoph Geisler

**Affiliations:** a GlycoBac LLC, Laramie, Wyoming, USA; b Department of Molecular Biology, University of Wyoming, Laramie, Wyoming, USA

## Abstract

Many vectors that are commonly used in the baculovirus/insect cell system (BICS) are derived from the *Autographa californica* multiple nucleopolyhedrovirus (AcMNPV) strain E2. To facilitate work with these vectors, we sequenced the E2 genome, compared it to that of the AcMNPV C6 strain, and found that they are very similar overall.

## GENOME ANNOUNCEMENT

The baculovirus/insect cell system (BICS) (1, 2) is used to produce relatively high levels of correctly folded recombinant proteins with eukaryotic posttranslational modifications for diverse applications (3–8). Baculoviral vectors used in the BICS are almost always derived from *Autographa californica* multiple nucleopolyhedrovirus (AcMNPV) strains C6 (vector including BacPAK/flashBAC, Bac-N-Blue, and BaculoGold) or E2 (vectors including BestBac, BaculoDirect [9], Bac-to-Bac [10], and MultiBac).

The AcMNPV C6 genome sequence was reported in 1994 (11), which provided valuable information for the production and screening of baculoviral vectors derived from this strain. In contrast, only partial sequences are available for AcMNPV E2. Thus, to facilitate the production and screening of baculoviral vectors derived from E2, we determined its complete genome sequence.

The E2 virus used for this purpose was derived from stocks originally acquired from the laboratory of Max D. Summers at Texas A&M University (12). The virus was amplified in Sf9 cells (13), viral genomic DNA was extracted from infected cells (14), and the DNA was subsequently sequenced using the Ion Torrent system (Invitrogen) by PrimBio. DNA sequences were assembled *de novo*, and the resulting contigs were assembled manually. Gaps were closed by Sanger sequencing of PCR amplimers, and the sequences of the larger homologous region (HR) repeat elements and questionable regions were confirmed by Sanger sequencing of PCR amplimers, some of which were cloned. Finally, the complete genome sequence was manually curated and annotated.

Overall, the AcMNPV E2 genome was colinear with and very similar to that of the C6 reference sequence (accession no. NC_001623.1), with the same genes and genetic elements distributed in the same order across a slightly larger genome (133,966 versus 133,894 bp). The difference in genome size was largely due to an additional repeat in the E2 homologous region 2 (HR2) element (15), which also represented the largest individual difference between the genomes. A comparison of the C6 and E2 HR2 elements also revealed further differences, including several insertions, deletions, and single nucleotide polymorphisms (SNPs). Another region of substantial genetic variation between E2 and C6 was found to be the HR4b element. These results are consistent with the previously reported observation that HRs are hot spots of genetic variability among baculovirus strains (16).

In addition to the differences noted above, we found several dozen SNPs and other changes between the E2 and C6 open reading frames (ORFs). In cases in which the E2 gene product differed from the C6 gene product, the E2 gene product was generally identical to homologues from other closely related baculoviruses, such as *Plutella xylostella* MNPV (17) or *Rachiplusia ou* MNPV (18). Furthermore, there were several instances in which the E2 sequence did not match the C6 whole-genome reference sequence but matched other partial C6 sequences in GenBank, indicating that the C6 reference sequence might be erroneous at those positions. This is in agreement with a report that the C6 reference sequence is incorrect in several regions, as determined by resequencing (19).

### Nucleotide sequence accession number.

The complete genome sequence of AcMNPV E2 was deposited with GenBank under accession no. KM667940.
